# Developing a Roadmap for Improving Neglected and Underutilized Crops: A Case Study of South Africa

**DOI:** 10.3389/fpls.2017.02143

**Published:** 2017-12-14

**Authors:** Tafadzwanashe Mabhaudhi, Vimbayi G. P. Chimonyo, Tendai P. Chibarabada, Albert T. Modi

**Affiliations:** Crop Science, School of Agricultural, Earth and Environmental Sciences, University of KwaZulu-Natal, Pietermaritzburg, South Africa

**Keywords:** champions, development, potential, utilization, value chain

## Abstract

Reports of neglected and underutilized crops' (NUS) potential remain mostly anecdotal with limited and often incoherent research available to support them. This has been attributed to lack of clear research goals, limited funding directed at NUS and journal apathy toward publishing work on NUS. The latter points also explain the lack of interest from emerging and established researchers. Additionally, the NUS community's inability to articulate a roadmap for NUS' promotion may have unintentionally contributed to this. The current study is a sequel to an initial study that assessed the status of NUS in South Africa. The objective of this follow-up study was then to (i) identify priority NUS, and (ii) articulate a strategy and actionable recommendations for promoting NUS in South Africa. The study identified 13 priority NUS, categorized into cereals, legumes, root, and tuber crops and leafy vegetables based on drought and heat stress tolerance and nutritional value. It is recommended that the available limited resources should be targeted on improving these priority NUS as they offer the best prospects for success. Focus should be on developing value chains for the priority NUS. This should be underpinned by science to provide evidence-based outcomes. This would assist to attract more funding for NUS research, development and innovation in South Africa. It is envisaged that through this roadmap, NUS could be transformed from the peripheries into mainstream agriculture. This study provides a template for developing a roadmap for promoting NUS that could be transposed and replicated among the 14 other southern African states.

## Introduction

Securing future food and nutrition security requires a paradigm shift from the current lock-in situation. According to Pahl-Wostl (2009), a lock-in situation arises when institutions and role players in decision making continue with a conventional approach, despite that it is no longer leading to desired outcomes. This analogy applies to the continuation of Green Revolution technologies, despite emerging consensus that they may not be capable to deliver on future food and nutrition targets. While the existing system of agriculture, which focuses on a few major food crops, may have succeeded in ensuring national food security in the past, its ability to continue doing so in the twenty-first century has been challenged (Mabhaudhi et al., 2016a) with calls for greater diversification of rural cropping systems. There are calls for a paradigm shift in agriculture to explore non-conventional pathways such as underutilized crops (NUS) as possible future crops (Massawe et al., 2015). This is premised on reports that NUS are adapted to a range of agro-ecologies, and may be nutrient dense and offer better prospects in marginal production areas (Mayes et al., 2012; Chivenge et al., 2015; Massawe et al., 2015).

Several NUS are drought and heat stress tolerant, resistant to pests and diseases, and adapted to semi-arid and arid environments (Mayes et al., 2012; Chivenge et al., 2015; Chimonyo et al., 2016; Chibarabada et al., 2017; Hadebe et al., 2017; Mabhaudhi et al., 2017). In addition, most NUS are nutrient dense (Padulosi and Hoeschle-Zeledon, 2004) and could be useful in diversifying diets (Mayes et al., 2012) and addressing micronutrient deficiencies in poor rural communities (Chibarabada et al., 2017; Govender et al., 2017; Hadebe et al., 2017). Thus, their promotion in marginal agricultural production areas could improve availability and access to nutritious food by rural people (Williams and Haq, 2000). Their promotion in rural areas could also create opportunities for rural economic development through the development of new value chains (Mabhaudhi et al., 2016a, 2017). Importantly, NUS, which include crop wild relatives (CWR), are an important germplasm resource for future crop improvements for beneficial traits such as nutritional value and abiotic and biotic stress tolerances (Castañeda-Alvarez et al., 2016). However, much of this reported potential is currently premised on anecdotal evidence with limited robust, empirical, and comparable information (Mabhaudhi et al., 2016b). Thus, there is a need to promote evidence-based approaches that can assist in developing policy and increased research funding to support NUS (Mabhaudhi and Modi, 2013).

In South Africa, organizations such as the Water Research Commission of South Africa and the national Department of Science and Technology have identified the potential of NUS to contribute to food and nutrition security and sustainable rural economic development. Together, they have funded research aimed at determining water use, drought tolerance, agronomy and nutritional value of several NUS. While this has been positive for NUS development, obstacles remain. Research funding remains skewed toward major crops that have a proven record of delivering on food security goals. The meager resources that are availed for NUS research are stretched across a wide range of species with focus on the start-up point of value chains. This translates into very little impact, which, in turn, challenges the ability to convince global funding agencies to fund underutilized crops' research. Targeting research and development as well as development of value chains for of a few NUS that have potential for success could provide some impetus to start challenging the dominance of major crops. Currently, there have been reports detailing NUS' potential; few have provided a roadmap on how to exploit this potential. Thus, there is a need to identify such high potential NUS and prioritize them and articulate strategy with actionable recommendations on how to exploit the potential of NUS.

A starting point would be to identify priority research NUS and develop plausible value chains. For South Africa and the region at large, this must be aligned to addressing present and future challenges such as climate change and food and nutrition security. Thus, focus should be on identifying those NUS that are drought and heat stress tolerant as well as being nutrient dense. The adaptation of NUS to niche ecologies, their role in diversification (increased ecosystem productivity), food and nutrition security and building resilience (Tilman and Snell-Rood, 2014) should be captured. This emphasizes the need for a context-specific roadmap that recognize the niche existence of NUS. Hence, the prioritization should include a range of crop/food groups and shift from the dominance of a few cereal and starchy crops. Therefore, the objectives of this study were to (i) identify priority NUS with potential for success, and (ii) articulate a roadmap for research, development and innovation for priority NUS in South Africa. While the focus of the study was on South Africa, it is expected that the outputs of the study would be applicable to the 14 other countries in southern Africa. Most of these countries share similar agro-ecologies, natural resources, agro-biodiversity, culture, and heritage.

## Methodology

### Research context

The current study is a sequel to an initial study on the status of NUS in South Africa, which recommended prioritizing NUS and developing a strategy to promote them (Mabhaudhi et al., 2017). The current study was structured into two stages, (i) identifying priority drought tolerant and nutrient dense NUS, and (ii) outlining a roadmap for promoting research, development and innovation for the identified NUS in South Africa. Details of these stages are outlined below.

**Stage 1: Prioritisation of underutilized crops**

A phased approach was used to develop a list of priority drought tolerant and nutrient dense NUS for South Africa. This included three phases, namely, (i) literature search, (ii) resource identification, and (iii) prioritization.

**Phase 1: Literature search**

A mixed method approach, which included combining quantitative and qualitative research or outcomes with process studies, was used. Emphasis was placed exclusively on identifying literature on NUS from South Africa. Briefly, the study initially identified the terms or key words commonly used to refer to underutilized crops in South African literature. The five (5) commonly used terms that were identified were; (i) underutilized crops, (ii) indigenous crops, (iii) traditional crops, (iv) neglected crops, and (v) orphan crops. This was based on common terminology identified in the initial study (Mabhaudhi et al., 2017). The terms were used to conduct online searches using Google® and Google Scholar®, Scopus, ScienceDirect and SpringerLink search engines. Articles of interest were those relating to South African literature hence the search was set to filter results to the country South Africa. Results were further filtered to only show results that featured at least one of the following exact words “underutilized/indigenous/traditional/neglected/orphan.” Thereafter, results were separated into pre- and post-2000 periods and further separated into scientific, public, and online publications. Scientific articles included research papers, theses, conference proceedings, and technical reports.

The initial literature search showed that, in South Africa, “traditional crops” was the most popular term with 236,000 hits, while “indigenous crops” was the least popular with 1,110 hits. When results were filtered to only show results that featured at least one of the exact words “underutilized/indigenous/traditional/neglected/orphan,” “neglected crops” became the most popular term with 112,000 hits.

**Phase 2: Resource identification**

The objective of Phase 2 was to use outputs of Phase 1 to identify (i) common underutilized crops, and (ii) research themes or focus areas. The resource identification was also developed as an initial step toward developing a tool for archiving information on and developing a database for underutilized crops research in South Africa.

- Crops that were identified included bambara groundnut (*Vigna subterranea* (L.), amaranth (*Amaranthus sp*.), bottle gourd (*Lagenaria siceraria*), maize landraces (*Zea mays*), cowpea (*Vigna unguiculata* (L.) Walp), sweet potato (*Ipomoea batatas*), taro (*Colocasia esculenta*), sword bean (*Canavalia gladiate*), black jack (*Bidens pilosa*), marama bean (*Tylosema esculentum*) jews mallow (*Corchorus olitorius*), spider plant (*Cleome gynandra*), pearl millet (*Eleusine coracana*), nightshade (*Solanum nigrum*), chinese cabbage (*Brassica chinensis*), cocoyam (*Xanthosoma spp*.), sunberry (*Solanum nigrum*), wild water melon (*Citrullus lanatus*), wild mustard (*Sinapis arvensis*), sorghum (*Sorghum bicolour*), sesame (*Sesamum indicum* L.), and tef (*Eragrostis tef*). It was noted that from the list of identified underutilized crops, not all of them have CWR in South Africa. However, despite most crops not being indigenous to South Africa, they have become traditional crops in that their cultivation has formed part of traditional cropping systems for more than 100 years, from which local landraces have derived (Mabhaudhi et al., 2017).
- Common research themes identified included nutrition, ecophysiology, agronomy, crop modeling, food security, seed quality, breeding, and biotechnology, perceptions, climate change, postharvest, genetic resources, medicinal properties, and commercialization. Concurrently, the amount of research per crop per research theme was tabulated. The objective of this parallel exercise was to provide an initial assessment of gaps in knowledge on identified NUS in relation to the thematic areas of research.

**Phase 3: Identifying priority underutilized crops**

The objective of this phase was to develop a list of priority drought tolerant and nutrient dense NUS for South Africa. Initially, the list of priority NUS for research in Africa proposed by Williams and Haq (2000) was used as a baseline for the current study and compared to NUS currently researched in South Africa. Thereafter, literature search on drought tolerance, heat stress tolerance and nutrient density was conducted *viz*. the initial list of NUS identified in Phase 2. The objective was to identify an initial priority list of NUS of interest in South Africa based on drought tolerance and nutrient density of different food groups (cereals, legumes, vegetables, roots, and tuber crops). Crops that were reported in the literature as being drought and heat stress tolerant and that were nutrient dense were identified. Based on this, a list of priority NUS was developed based on food groups of cereals, legumes, leafy vegetables and root, and tuber crops.

**Stage 2: Developing a roadmap**

The development of the strategy involved a three-step approach, namely, (i) a diagnosis of challenges, obstacles and opportunities, (ii) priority setting and developing a framework for addressing challenges, and (iii) developing a guiding framework outlining coherent actions and activities for future funding of RDI on NUS for South Africa. This followed a conceptual and theoretical framework approach (De Houwer, 2007; Sinclair, 2007). The conceptual framework was akin to an inductive approach and synthesized existing concepts concerning research, development and innovation (RDI) relevant for NUS in South Africa. Briefly, a theoretical framework is a deductive approach, which assesses existing literature. This was used to identify key focus areas for RDI on NUS for South Africa.

## Results and discussion

### Resource identification and identifying research gaps

The use of many terms used to refer to NUS confirmed earlier reports of a lack of a consensus definition (Mabhaudhi et al., 2017). Previous reports have attributed the scattered nature of NUS research outputs to the lack of a consensus definition (Chivenge et al., 2015; Mabhaudhi et al., 2016c, 2017). The emerging trend has been toward the use of neglected and underutilized crop species (NUS). In terms of research outputs, the literature showed an average 10-fold increase in research on NUS during the post-2000 period. This is consistent with anecdotal reports of emerging interest on NUS as part of solutions to food production and security in the twenty-first century.

Results of resource identification showed that the most researched themes on underutilized crops were nutrition and ecophysiology with 15 and 14 publications, respectively (Table 1). Themes that featured prominently (>4 publications) were agronomy, crop modeling and genetic resources, while climate change, breeding and biotechnology, peoples' perceptions, medicinal properties and commercialization featured to a limited extent (<3 publications). This confirmed previous reports that have identified crop improvement and development of value chains or marketing as key impediments to the promotion of NUS is South Africa and elsewhere (Chibarabada et al., 2017). While much work has been done addressing tolerance to abiotic stresses (drought and heat) and nutritional value, there is a need to focus on crop improvement for NUS and developing their value chains.

**Table 1:** List of underutilized crops and the number of times each crop was researched under a theme based on the South Africa resource identification search.

**Crops**	**Total**	**Nutrition**	**Ecophysiology**	**Agronomy**	**Crop modelling**	**Food security**	**Seed quality**	**Popular articles**	**Breeding**	**Perceptions**	**Climate change**	**Post-harvest**	**Genetic variation**	**Medicinal properties**	**Commercialisation**	**Biotechnology**
^*^ALVs	24	11	1	1	1	13	–	–	–	3	–	–	–	–	1	–
^**^ICs	15	1	3	2		7		4		–	1		1	–	1	–
B.groundnut	14		5	2	1		4		1	–	1			–	–	–
Amaranth	4	–	–	2	–	–	1	–	–	–	–	–	1	–	–	–
Bottle gourd	2	–	–		–	–	2	–	–	–	–	–	–	–	–	–
Maize landraces	4	–	–	1	–	–	3	–	–	–	–	–	–	–	–	–
Cowpea	6		1	2	1	–			2	–	–	–	–	–	–	–
Sweet-potato	3	1	–	–	–	–	2	–	–	–	–	–	–	–	–	–
Taro	12	1	4	2	2		–	–	–	–	1	1	1	–	–	–
Sword bean	1		–	–	–	1	–	–	–	–	–	–	–	–	–	–
Black jack	1		–	–	–	–	–	–	–	–	–	–	–	1	–	–
Marama bean	2	2	–	–	–	–	–	–	–	–	–	–	–	–	–	–
Jews Mallow	1	–	–	1	–	–	–	–	–	–	–	–	–	–	–	–
Spider plant	1	–	–	1	–	–	–	–	–	–	–	–	–	–	–	–
Finger Millet	1	–	–	–	–	–	–	–	–	–	–	–	–	–	–	1
Nightshade	1	–	–	–	–	–	–	–	–	–	–	–	1	–	–	–
Chinese Cabbage	1	1	–	–	–	–	–	–	–	–	–	–	–	–	1	–
Cocoyam	1	1	–	–	–	–	–	–	–	–	–	–	–	–	–	–
Sunberry	1	–	–	1	–	–	–	–	–	–	–	–	–	–	1	–
Wild mustard	1	–	–	–	–	–	1	–	–	–	–	–	–	–	–	–
Sorghum	3	–	1	–	1	–	–	–	–	–	–	–	–	–	–	1
Sesame	1	1	–	–	–	–	–	–	–	–	–	–	–	–	–	–
Tef	1	1	–	–	–	–	–	–	–	–	–	–	–	–	–	–
Wild water melon	3	1	2	–	–	–	–	–	–	–	–	–	–	–	–	–
Number of articles^†^		**15**	**14**	**6**	**4**	**11**	**11**	**5**	**2**	**2**	**3**	**1**	**4**	**2**	**2**	**2**

* *ALVs = Indigenous leafy vegetables and refers to articles that have addressed indigenous, underutilized, wild, and traditional vegetables with no particular focus to any single leafy vegetable*.

** *IC = Indigenous crops and refers to articles that have addressed indigenous, underutilized, wild, and traditional crops with no particular focus to any single crop*.

† *Publications referred to include all the documented publications (publications, reports, conference proceedings and online articles)*.

*The themes were identified from the initial literature search and show the wide scope of research that has been undertaken on neglected and underutilized crops in South Africa. The table also highlights areas were research has been lagging, i.e., gaps in existing knowledge on underutilized crops in South Africa*.

With respect to crops, vegetable crops have received the most research attention with amaranth being the most researched vegetable crop. The high research outputs for vegetables links well with their perceived role in addressing micronutrient deficiencies in diets of poor rural people (Govender et al., 2017). Bambara groundnut and cowpea were the most researched grain legumes with 14 and 6 publications, respectively. The high number of publications on bambara groundnut also aligns with international efforts being driven by the Bambara Groundnut Network (BamNetwork) to promote bambara groundnut as an exemplar underutilized crop (www.bambaragroundnut.org). Among the cereal crops, maize landraces and sorghum were the highest with four and three publications, respectively. With respect to root and tuber crops, taro received the most research attention (12 publications) followed by sweet potato (three publications) (Table 1). The successful commercialization of taro in South Africa (Modi, 2003; Agergaard and Birch-Thomsen, 2006) could be associated with the increased research outputs. Research on sweet potato has emerged recently, mostly due to promotion of orange-fleshed sweet potatoes as a source of beta carotene (Motsa et al., 2015).

### Identifying priority underutilized crops

A major reason for the low investments in NUS research is the perception that they offer less returns on investments compared to the major crops (Nelson et al., 2004). The initial study by Mabhaudhi et al. (2017) proposed that this hurdle could be overcome by identifying a few specific NUS with traits that are useful such as drought and heat stress tolerance and nutrient density, that show certain advantages over major crops and that had prospects for success. A major objective of the current study was therefore to develop a list of priority NUS for South Africa. As an initial step to identifying priority NUS for South Africa, the identified underutilized crops from local research (Table 2) were compared to Williams and Haq's (2000) list of priority underutilized crops for Africa (Table 2). Results of the comparison of underutilized crops listed by Williams and Haq (2000) to those currently researched in South Africa showed a positive match for 12 locally researched underutilized crops (Table 2).

**Table 2:** A comparison of crops listed as priority underutilized crops for Africa by Williams and Haq (2000) and the list of underutilized crops currently researched in South Africa.

	**Common name**	^*^Priority crops for Africa	^**^Currently researched in South Africa	**Drought tolerance**	**Heat stress tolerance**	**Publications**
Cereals	Sorghum		X	√		3
	Finger Millet	X	X			1
	Tef		X		X	1
	Maize landraces		X			4
	Barnyard grass	X				
Legumes	Bambara nut	X	X	√		14
	Lablab	X	X			1
	Pigeon pea					
	Sword bean	X	X		X	1
	Cowpea		X	√		6
	Velvet bean	X				
	Marama bean		X			2
Root and tubers	Taro	X	X			
	Sweet-potato		X			
	Cassava	X				
	African yam bean	X				
	Cocoyam	X	X			
Vegetables	Bottle gourd	X	X			2
	Black jack	X	X	√		1
	African Eggplant	X				
	Jews Mallow	X	X	√		1
	Roselle	X				
	Spider plant		X	√		1
	Amaranth	X	X	√		4
	Nightshade	X	X	√		1
	Chinese Cabbage	X	X	√	X	1
	Sunberry		X			1
	Wild mustard		X			1
	Wild Water Melon	X	X			3

* *Obtained from Global Research on Underutilized Crops an Assessment of Current Activities and Proposals for Enhanced Cooperation (Williams and Haq, 2000)*.

** *Includes Crops obtained from the database created from this study on underutilized crops that are currently featuring in South African research*.

*√Based on South African research*.

*The identified underutilized crops are then compared with regards to drought and heat stress tolerance based on available South African and international literature*.

*X Under the columns, “priority crops” and “currently researched in South Africa” denotes that these crops have been listed. Under heat stress tolerance column the X denotes that available research shows that these crops are not tolerant to heat stress*.

Among the cereal crops, only finger millet featured on both lists while for legumes, bambara groundnut, sword bean, and lablab featured on both lists. Taro and cocoyam were the two root and tuber crops that featured on both lists. The exclusion of locally imports NUS such as cowpea, pigeon pea, sorghum, tef, sweet potato, and maize landraces was attributed to varying global definitions of NUS and the reference to “where, when and by who” (Padulosi, 2006) that are often applied when defining underutilized crops. This inadvertently creates discrepancies in national inventories of NUS (Hadebe et al., 2017). In a recent review on cereals, Hadebe et al. (2017) argued that although sorghum was generally considered to be an established crop because of its importance in several parts of sub-Saharan Africa, it was still underutilized relative to its potential. This is the case for similar crops such as tef, which are important in other African countries but locally underutilized.

There were also crops that featured on Williams and Haq's (2000) list of Africa's priority underutilized crops which did not appear on South Africa's list of NUS. These included barnyard grass, velvet bean, cassava, African yam bean, African eggplant and roselle (Table 2). With regards to crops such as cassava, while it is an important NUS and staple in parts of sub-Saharan Africa, its levels of utilization in South Africa remain extremely. It may also be that some of these crops are already being researched in South Africa but did not come up in the literature search that was conducted. This is because a lot of work on NUS remains unpublished and scattered.

Consistent with the results of the resource identification, the majority of crops on both Williams and Haq's (2002) and the South Africa list were indigenous leafy vegetables (bottle gourd, blackjack, jews mallow, amaranth, nightshade, wild water, melon and Chinese cabbage) (Table 2). This confirmed the dominance of indigenous leafy vegetables within the family of underutilized crops. Modi (2015) also reported that several of these indigenous leafy vegetables had significant nutritional potential to contribute to the diets of rural people. Modi (2015) further noted that indigenous leafy vegetables had previously formed an important component of rural diets but were now on the decline. However, despite their relatively higher research outputs compared to other NUS, indigenous leafy vegetables remain underutilized due to constraints such as perception, processing, distribution, and marketing, as well as lack of information on their nutritional value and bioavailability.

To further identify underutilized crops of interest in South Africa, we conducted a literature search for drought and heat stress tolerance as well as nutritional composition for NUS from South Africa (Tables 2, 3). The aim was to identify crops that exhibited qualities of drought and heat stress tolerance (Table 2) and were nutrient dense (Table 3). Based on these criteria, 13 underutilized crops, split into cereals, legumes, root and tuber crops and leafy vegetables, were identified as priority drought tolerant and nutrient dense NUS (Table 4). Consistent with the trend in research outputs, indigenous leafy vegetables dominated the list. Several authors have previously reported on the adaptation (drought and heat stress tolerance) and nutritional value of several of the cereals (Hadebe et al., 2017), legumes (Chibarabada et al., 2017), root and tuber crops (Mabhaudhi and Modi, 2013; Motsa et al., 2015) and indigenous leafy vegetables (Oelofse and van Averbeke, 2012; Modi, 2015).

**Table 3:** A comparison of the nutritional value (based on raw 100 g portion) of selected underutilized crops listed as priority crops for Africa by Williams and Haq (2000) and selected underutilized crops identified for South Africa.

	**Common name**	**Energy (kcal)**	**Protein (g)**	**Fat (g)**	**Fibre (g)**	**Ash**	**CHO (g)**	**Ca (mg)**	**P (mg)**	**Na (mg)**	**Mg (mg)**	**Cu (mg)**	**Zn (mg)**	**Fe (mg)**
**Cereals**	Maize landraces	339	13.7	2.47	2.7	1.78	71	34	508	2	3.01	0.55	4.16	3.01
	Sorghum	329	10.9	3.2	2.3	1.6	73	27	215	4	103	0.3	1.5	2.6
	Finger millet	363	11	5	2.2	1.9	69	25	–	–	–	–	–	–
	Tef	367	13	2.4	8	2.49	73	0.19	13	0.01	354.18	–	37.30	50.78
**Legumes**	Bambara	386.32	21.85	6.9	3.42	3.6	53.39	219.26	266.1	11.9	2.6	0.41	7.9	7.02
	Cowpea	357.1	24.7	4.8	2.8	4.2	51.76	180.46	310.94	107.24	1.74	9.9	5.3	4.9
	Lablab	117	26.86	0.27		3.96	67.23	–	8	–	–	–	0.38	0.76
	Sword Bean	1560.3	28.39	7.84	8.23	5.63	49.91	–	–	–	–	–	–	–
	Marama bean	477	34.71	40.06	3.94	3.19	14.07	241	454	63.75	274.5	1.04	6.2	3.95
**Root and tuber crops**	Taro	102	7.79	0.65	3.01	2.44	86.11	55	1.6	–	–	–	1.67	–
	Sweet potato	86	1.6	0.1	3.0	1.05	20.1	30	47	55	25	3	249	0.42
	Cocoyam	112	1.5	0.2	4.1	–	26	–	–	–	–	–	–	–
**Vegetables**	Amaranth	49	4	0.2	2.87	3.42	7.86	1686	487	347	82	3	56	25
	Nightshade	55	3	0.6	2.42	2.24	9.03	2067	478	431	3	6	23	85
	Black jack	39	5	0.6	2.92	2.82	3.72	1354	504	290	21	10	22	17
	Jews Mallow	392	20.90	5.20	45.61	–	55.50	1760	490	801.20	15.50	11.30	12.40	53.30
	Wild mustard	26	2.7	0.2	1.1	1.4	4.9	–	–	–	–	–	–	–
	Bottle gourd	14	0.62	0.02	0.5	0.5	3.39	26	13	2	0.089	0.034	0.70	0.20
	Chinese Cabbage	21	9	1	1.0	1.4	22	152	32	29	42	0.07	0.30	1.4
	Sun–berry	38	5.8	0.8	1.4	8.8	5.0	442	75	–	–	–	–	4.2
	Spider plant	–	7.7	0.9	1.6	3	6.4	434	12	33.6	86	0.46	0.76	11
	Wild water melon	296	3.5	0.4	3.8	1.66	13.1	212	119	9	59	0.20	0.74	6.4

*The table highlights the varying nutrient density of underutilized crops and contributed to the development of priority underutilized crops for South Africa*.

**Table 4:** List of thirteen (13) priority drought tolerant and nutrient dense underutilized crops for South Africa.

	**Common name**	**Scientific name**
Cereals	Sorghum	*Sorghum bicolor*
	Tef	*Eragrostis tef*
Legumes	Bambara groundnut	*Vigna subterranea* (L.)
	Lablab	*Lablab purpureus* (L.) Sweet
	Cowpea	*Vigna unguiculata* (L.) Walp
	Marama bean	*Tylosema esculentum*
Root and tubers	Taro	*Colocasia esculenta*
	Sweet-potato	*Ipomoea batatas*
Leafy vegetables	Jews mallow	*Corchorus olitorius*
	Spider plant	*Cleome gynandra*
	Amaranth	*Amaranthus sp*.
	Nightshade	*Solanum nigrum*
	Wild water melon	*Citrullus Lanatus* L.

*The priority underutilized crops were identified based on existing knowledge in the literature with regards to drought and heat stress tolerance, and nutritional value, and further categorized into four food groups (cereals, legumes, root and tuber, and leafy vegetable crops)*.

The approach used to prioritize NUS was linked to addressing the gap between sufficient food supply and nutritional requirements and the demand to closely link increasing food production with nutritional outcomes (Mabhaudhi et al., 2016a). This is of importance to South Africa which faces challenges related to water scarcity, increasing population and increasing levels of malnutrition (Govender et al., 2017). The paradigm shift to a nexus thinking (water-food-nutrition and agriculture-environment-health nexus) provides some initial insight on research issues to be targeted in the future.

### Developing a strategy for promoting underutilized crops for South Africa

#### Considerations for the NUS strategy

Following from the exercise in prioritization, the next stage was articulating a strategy or roadmap for promoting the identified priority NUS. The primary objective was to identify priorities for research, development and innovation (RDI) for NUS that would guide future funding for NUS at a national level. Secondly, there was a need to ensure that the proposed strategy was aligned to existing national development priorities while also advocating for paradigm shifts in policy. The proposed roadmap also complements the existing approach to funding research on NUS that was developed by Water Research Commission of South Africa which emphases on (i) drought and heat stress tolerance, (ii) water use and nutritional value, and (iii) nutritional water productivity (Backeberg et al., 2010; Backeberg, 2014). The novelty in the proposed roadmap is linking sustainably increasing food production to nutrition and health outcomes (agriculture-environment-health nexus) and focus on a few priority and exemplar NUS which should translate to positive success stories on NUS.

**Figure 1:**
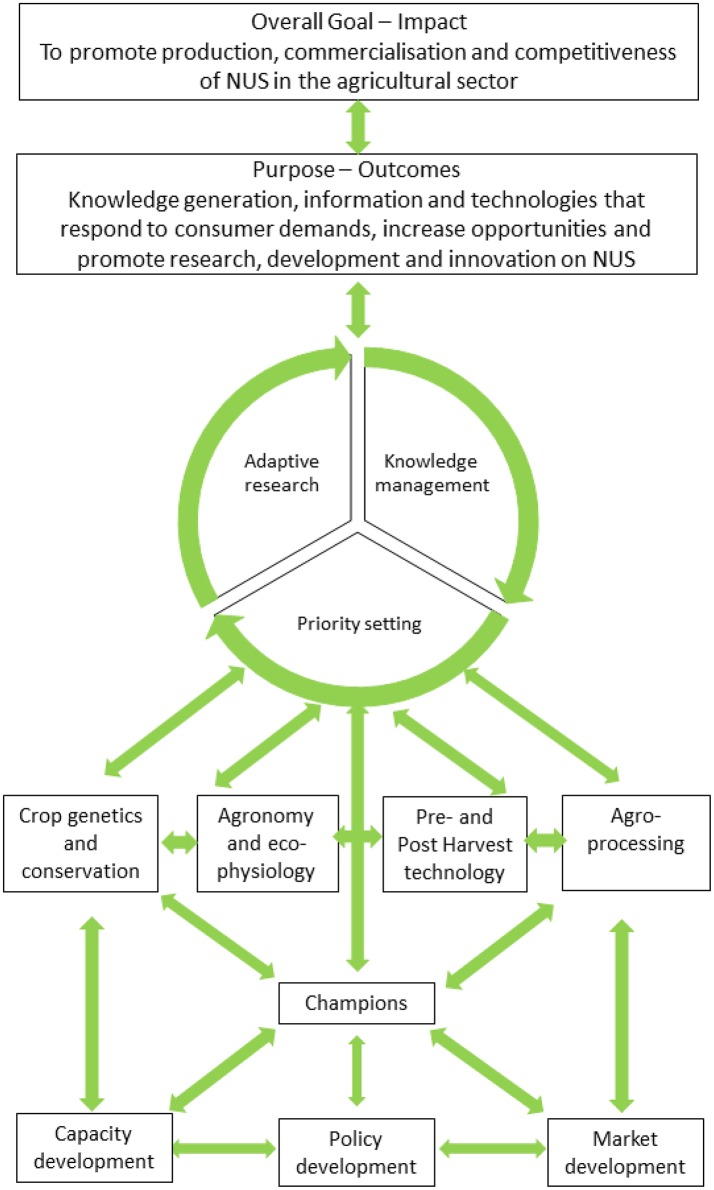
A schematic illustration for research, development and innovation (RDI) strategy for promoting underutilized crops in South Africa. Briefly, the framework promotes continuous priority setting, adaptive management and knowledge management as drivers of RDI along the value chain. The central role of champions in feeding into this RDI and taking outputs forward into human capacity development, policy, and market development is also highlighted.

The strategy is to consolidate gains already made through identifying research outcomes and existing research gaps and opportunities. The proposed strategy also attempts to set new priorities for funding that offer best prospects for success. The strategy articulates a series of actionable activities that provide a roadmap toward the desired paradigm shift for NUS research and development. This approach covers not only conventional research but also the use of innovation platforms, policy, markets, advocacy, knowledge management, and the involvement of a broad base of stakeholders (Figure 1). It is envisaged that through this strategy, NUS could be transformed into playing a key role in addressing pressing challenges related to food and nutrition security under water scarcity, informing climate change adaptation strategies and addressing the poverty-unemployment-inequality nexus. This can only be achieved if the translation of NUS into notable commercial successes is underpinned on RDI across the research value chain. As a result, the strategy places emphasis on being adaptive, hence, capable of responding to changing global paradigms and priorities. A key aspect of the strategy would be to ensure proper knowledge management of all information generated on NUS, which would contribute to the goal of establishing a database for NUS.

#### Adaptive research

Within smallholder farming systems, agricultural production is complex owing to varying levels of socio-economic and biophysical constraints and their interaction. Thus, promotion of any new technology should not assume a “one size fits all” approach. In the past, such assumptions have resulted in low adoption rates of imported technologies that had achieved successes under different spatial and temporal conditions. The successful promotion of NUS requires that RDI prioritize end users' needs as well as norms and values; this should be a key element to NUS' development. These are often inextricable to the intrinsic value that NUS hold within the communities that have preserved and still utilize them. In this regard, several inter-related components such as (i) development of appropriate technologies, (ii) dissemination to end users, and (iii) diagnosis of impacts, and (iv) redefining and/or customizing technologies are key to promoting NUS.

Adaptive research aims at devising site specific dynamic technology packages for increasing agricultural production (Lindenmayer and Likens, 2009). It helps to translate the results of research into suitable forms before disseminating it to farmers, making research outputs more relevant to their local agro-climatic and socio-economic conditions. Adaptive research bridges the gap between research findings and farmer and extension achievements and aspirations and continuously consolidates knowledge on the subject matter. This ensures that technologies remain relevant regardless of changing conditions. One of the main goals of the RDI strategy plan for NUS focusses on adaptive research. For adaptive research to be a core pillar within the strategy, information should be readily available and accessible in a format suitable for the different end users. An additional feature of the strategy framework would be to promote a system for knowledge management, which reflects current initiatives to develop dynamic and resilient protocols for the promotion of NUS in South Africa.

#### Knowledge management

Knowledge management is a discipline and a process that promotes an integrated approach to identifying, capturing, evaluating, retrieving, and sharing information. The process of knowledge management can also be used to simplify the complexity of documenting and sharing knowledge regarding NUS among stakeholders and could help align strategies, methods, and technologies with different development agendas. Knowledge management could help to identify knowledge gaps and research priorities pertaining to NUS; improving the overall focus of the strategy. The definition of ‘knowledge’ itself is an on-going process (Berkes et al., 2000; Moller et al., 2004) with different relevance and perspectives to its users and is an important aspect within the strategy for NUS. This is consistent with the adaptive capacity of the strategy.

Currently, major crops are well-endowed with coherent knowledge (scientific and indigenous) and this has contributed to their dominance. Contrary to this, many NUS have neither the coherent body of work, nor comprehensive research concerning how such a body of knowledge could or should be funded, assembled and put to work in raising the status of NUS (Mabhaudhi et al., 2016d). It is increasingly recognized that promoting new technologies can be enhanced by incorporating knowledge from multiple sources, including the perspectives of researchers, farmers and other stakeholders; the same should apply for NUS. Indigenous communities have been identified as the main custodians of knowledge regarding NUS. Much knowledge pertaining to the utilization and intrinsic value associated with NUS remains hidden in the indigenous knowledge (IK) of these communities. There is need to capture relevant IK and incorporate it in to NUS' RDI; this would help to steer away from top-down prescriptive development strategies. Indigenous knowledge, integrated with scientific knowledge, is an essential resource for establishing competitive and comparative advantages for NUS in modern day agriculture. Appropriate knowledge management mechanisms are, therefore, required to more efficiently harness these different sources of knowledge and facilitate their broader adoption, promotion, dissemination and application (Ison and Russell, 2007). This would also address the paradox regarding which or whose, values should guide priority-setting decisions and how these values should inform decisions on promoting NUS.

#### Priority setting

Research, development and innovation on NUS is currently under-developed. Consequently, the scale and complexity of focus areas is large making it challenging to develop a well-articulated strategy. A major focus of the proposed strategy is to consolidate gains already made, establish new priorities that cater for specific NUS with potential and ensure that future research targets all points of the value chain. It is widely recognized that priority setting is a critical step for any efficient and coherent strategic plan. Within the context of this strategy, priority setting can be defined as a process of identifying activities that offer the best value and prospects of success for RDI for NUS. Priority setting aims to appraise and harmonize RDI activities that will allow for the mainstreaming of NUS into current farming and cropping systems. Moreover, it is also envisioned that it will assist with the formulation of specific policies for NUS, strengthen investment and encourage equitable resource allocation for RDI. Hence, the process of priority setting should:

- be consistent with the research value chain,
- permit continuous monitoring, evaluation, and assessment to allow for an adaptive process, and
- reflect the needs of all stakeholders along involved in the value chain.

**Figure 2:**
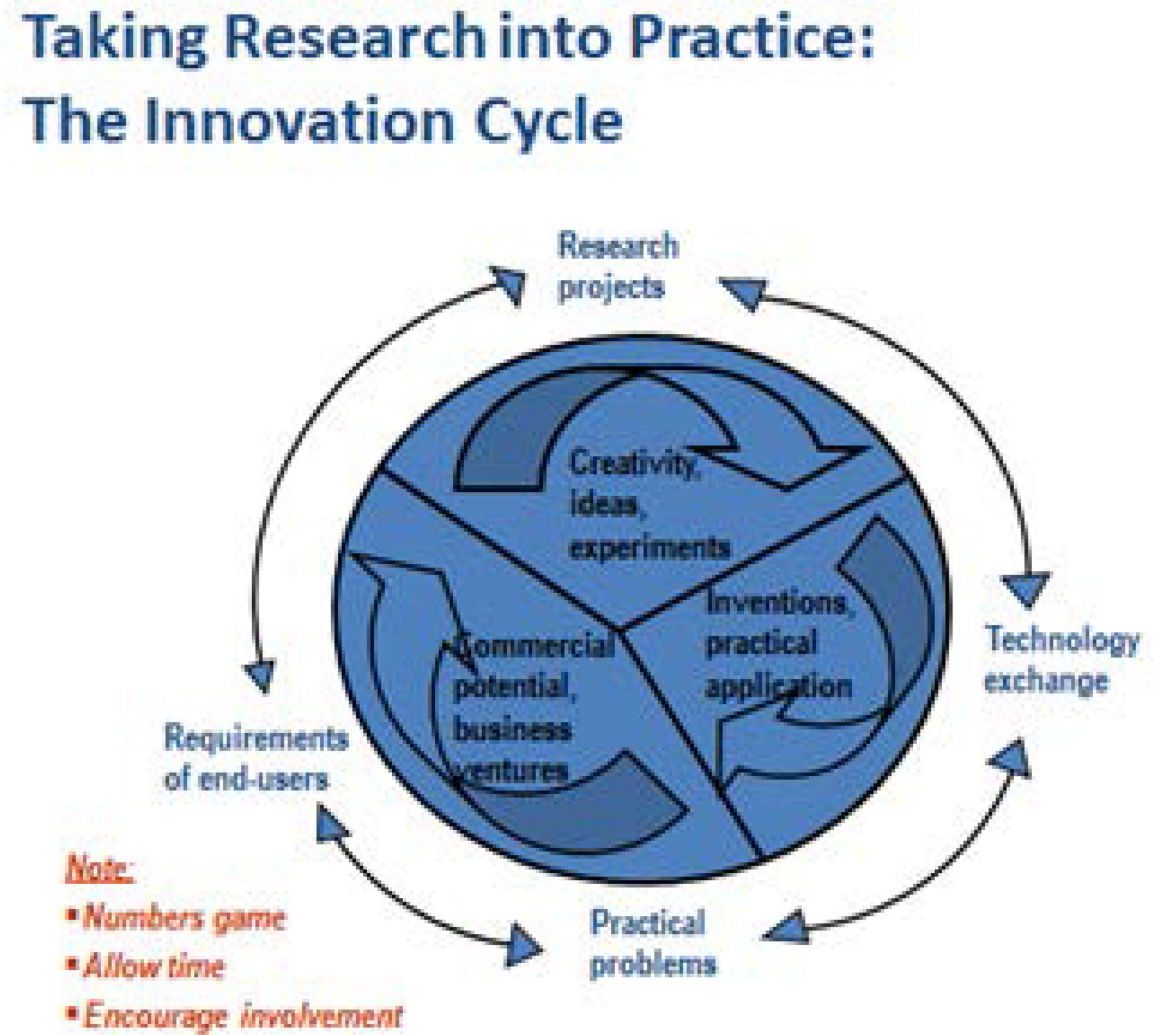
The current innovation cycle for the Water Research Commission (WRC). The WRC's innovation cycle provides an example of adaptive research similar to the one proposed in the current roadmap for promoting neglected and underutilized crops in South Africa. (Source: 12).

**Figure 3:**
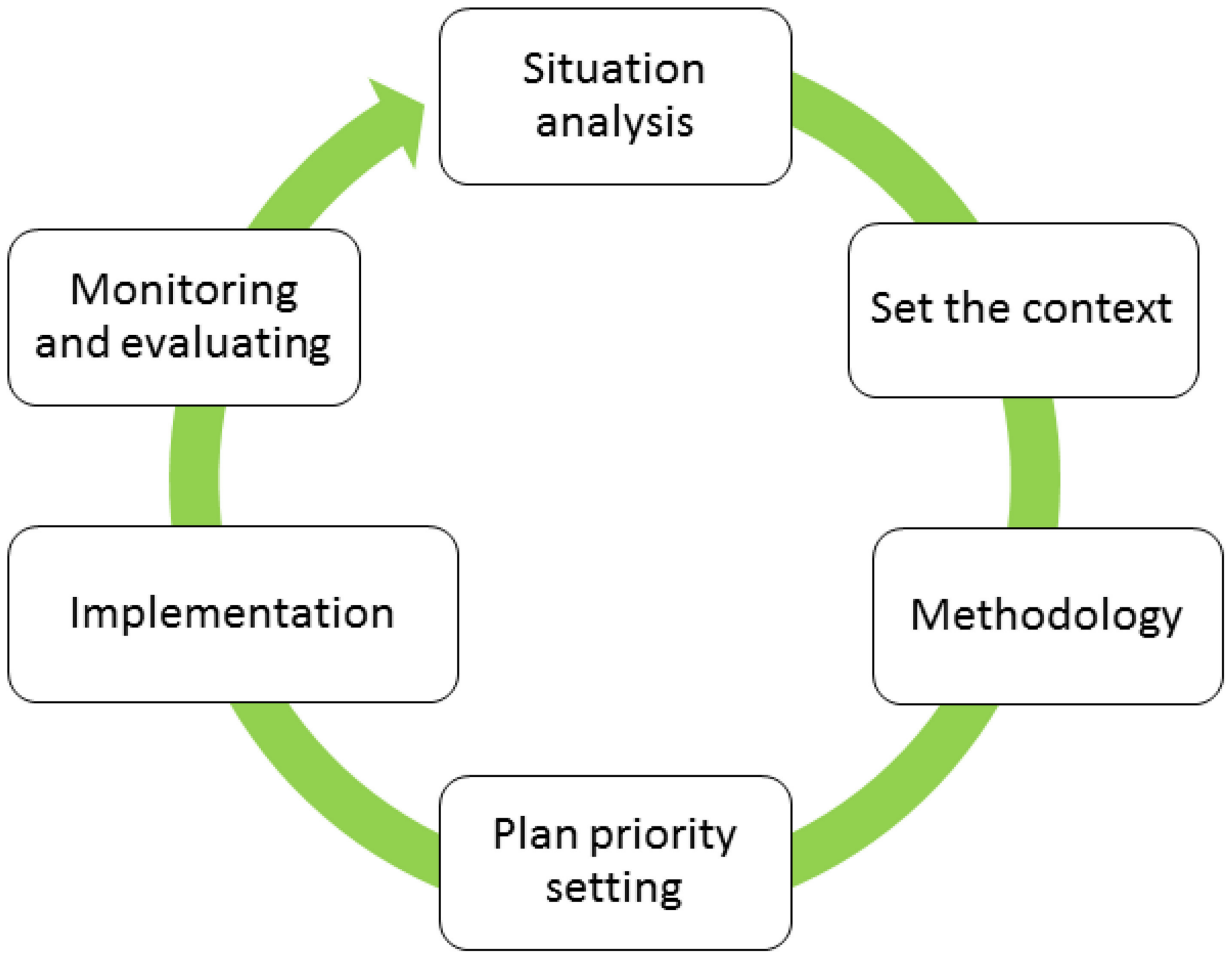
An illustration of the processes in priority setting for the research, development and innovation roadmap for neglected and underutilized crops in South Africa. The process starts with a situation analyses, which then leads to establishing the context and developing appropriate methodologies. This is followed by setting the priorities and developing a plan for implementation, monitoring and evaluation.

These attributes are consistent with the WRC's innovation cycle (Figure 2) and the six-step priority setting process outlined in Figure 3 and the development of an adaptive strategy for NUS.

### Policy

#### Alignment with existing policies and sustainable development agenda

Implementation of the strategy for promoting NUS can only be successfully if it is supported by, associated with and aligned to international, regional and national policies. Overall, the promotion of NUS offers strong linkages with Sustainable Development Goals (SDGs) 1, 2, 3, 8, and 15. It also speaks to the Southern African Development Community's (SADC) Regional Indicative Strategic Development Plan (RISDP) (RISDP, 2015), which provides a guiding framework for poverty eradication; sustainable food security; and human and social development as priority intervention areas. The roadmap for promoting NUS is also consistent with South Africa's National Development Plan (NDP)—Vision 2030 (National Planning Commission, 2013), the 2015 Nine Point Plan, the New Growth Path and Outcomes 4, 7, and 10 of the Medium Strategic Framework, which supports creation of employment opportunities, rural economic development and environmental conservation. In addition, the strategy for promoting NUS is aligned to key national policies such as The Integrated Growth and Development Planning (IGDP) (2010); National Department of Agriculture, Forestry and Fisheries (DAFF) Strategic Plan for 2016–2020; National Food Security Production Programme; and the National Policy on Food and Nutrition Security (2014). All these policies emphasize on the need to improve smallholders' participation in mainstream agriculture and the use of sustainable agricultural techniques for improved household food and nutrition security.

However, while there are demonstrable linkages and alignment to existing policies, the challenge has always been on raising awareness of the potential benefits of NUS, increasing research funding allocated to NUS, and demonstrating practically how NUS can contribute to sustainably increasing food production, addressing food and nutrition security, and creating new opportunities for growth and employment in rural areas. The roadmap outlined in this study will address these challenges through (i) targeting a few priority NUS with potential for success, (ii) systematically building the body of available empirical information on these selected crops across the entire value chain, and (iii) focussing on nexus approaches that demonstrate capabilities to address cross-cutting issues.

#### Gaps in existing policies

While NUS's promotion shows alignment to existing policies, the opposite is not true. Existing policies do not explicitly identify NUS as a viable option for achieving policy objectives. The inclusion of NUS within mainstream agriculture requires that the existing institutional and policy environment recognize the opportunities that NUS offer for rural economic development. The outputs of targeted research on identified priority NUS and demonstrated successes will feed into developing a policy framework that (i) encourages a stable and supportive macro- and micro-economic and regulatory environment for NUS, (ii) promotes capacity development needed for NUS RDI, (iii) provides sufficient financial and material support for NUS RDI, and (iv) promotes innovation of technologies linked to agro-processing for NUS.

Increased availability of empirical information on NUS could be used to advocate for updating and/or strengthening existing policies so that they can be explicit on the role of South Africa's agro-biodiversity—NUS. For example, the strategic grain reserves system, which currently only prioritizes maize and wheat, could be revised so that there is inclusion of a broader set of crops along the mentioned categories of cereals and legumes crops. Such inclusion would also address dietary diversity through the formal recognition of the broader set of crops. Another example would be to ensure that cross cutting issues such as the water-energy-food nexus, poverty-unemployment-inequality nexus, water-food-nutrition-health nexus and agriculture-environment-health are mainstreamed into new policies that are currently being developed. Through such mainstreaming, NUS could be more widely recognized for the different roles that they can also play.

#### Champions

It is important to note that while the support of policy and a well-articulated strategy is critical for the success of RDI for NUS, much more is required to change the existing paradigms for NUS. There is a need to identify and support researchers and/or institutions that have shown a demonstrated record of accomplishment and commitment to advancing the status of NUS in South Africa. In this regard, the agenda places emphasis on the role of “champions” in supporting the strategy and involvement in developing future research priorities. While there are international centers focussing on NUS development such as Bioversity International (www.bioversityinternational.org) and Crops For the Future (www.cffresearch.org), these have limited resources. There is a need to establish and support a national or regional center of excellence dedicated to driving research on NUS. While this may sound ambitious, this option may offer advantages in terms of (i) nurturing capacity needed for underutilized crops RDI, (ii) coordinating the implementation of the strategy through, (iii) establishing national, regional and international partnerships, and (iv) attracting research funding to drive implementation of the strategy. Importantly, the establishment of such a center of excellence would create continuity, which often is the primary challenge in the case of ‘champions.’ This would be consistent with the regional strategy on developing centers of excellence for key strategic areas.

### Recommendations for future research and funding

The initial study by Mabhaudhi et al. (2017) on appraising the status of NUS in South Africa identified the following primary research gaps: (a) land use classification, (b) crop improvement and agronomic information, (c) crop ecophysiology, (d) post-harvest handling and storage, (e) nutritional content, (f) marketing, (g) product development, and (h) documentation of indigenous knowledge. These were tailored to address the various points within a value chain. These should form the basis of any future research and should be targeted for funding. Furthermore, the following activities should be targeted for research funding in the short-medium term:

- *Crop improvement through biotechnology*. According to Mayes et al. (2012), following from identification of priority NUS, the next step is to improve the genetics of the crops. Although, NUS possess many beneficial traits, they are generally low yielding, which makes them unattractive to farmers and developmental agents (Nelson et al., 2004). Increasing yield potential in major crops has largely been attributed to aggressive breeding and biotechnology programmes. There is also a need to employ such techniques to improve yield potential of NUS. Advances in plant genomics now provide breeders advanced molecular and bioinformatic tools that allow the study of the whole genome to accelerate breeding efforts such as genome sequencing. Research should focus on sequencing the genomes of priority NUS and applying advanced translational techniques using model crops (Cannon et al., 2009). Plant genomics could also be used to reveal new landscapes for NUS across different agro-ecological zones within South Africa and across the region. Underutilized crops and the sub-set of CWR, are an important germplasm resource for future crop improvement for nutrient dense and stress tolerant crops (Castañeda-Alvarez et al., 2016). Thus, genetic sequencing of NUS will aid in identifying genes that confer such beneficial traits that could be used for crop improvement of other species.
- *Linking increasing NUS production to nutrition and health outcomes*. Consistent with the paradigm shift needed in agriculture, the development of NUS should seek to address the agriculture-environment-health nexus as this is where they have the most potential to make impact. Currently, reports of their perceived nutritional value and health benefits are mostly anecdotal (Mabhaudhi et al., 2016c). This should include research on nutrient content, nutritional yield and water productivity, and bio-availability of nutrients in NUS. Research that links increasing production and/or yields on NUS to improved human health and nutrition will contribute to empirically addressing widely held perceptions of the associated health benefits of consuming NUS.
- *Climate change adaptation*. Much of the emerging interest on NUS also focus on their potential to contribute climate change adaptation (Massawe et al., 2015). However, other than selected reports of drought and heat stress tolerance, there is limited research confirming their suitability to future climates. For the priority NUS, research should focus on assessing climate change impacts on their production for selected bio-climatic regions, with a focus on fitting them into semi-arid and arid areas.

## Conclusion

The first step to developing a roadmap for NUS requires that we identify and prioritize a few NUS with potential to succeed. Thirteen (13) priority NUS were identified across different crop categories, based on their drought and heat stress tolerance and nutritional value; this is the first such list for South Africa. Research funding should then be directed toward priority research areas that have the greatest capacity to translate into notable gains. This includes crop improvement, linking food production to nutrition (agriculture-environment-health nexus) and climate change adaptation. This should be underpinned by human capacity development and knowledge management (integrating indigenous knowledge) adaptive priority setting to ensure sustainability. The new knowledge generated, and the success stories should be used to influence existing and new policies to advocate for increased allocation of research funding for NUS. In the long-term, the promotion of NUS should adopt a socio-ecological approach that integrates linking food production to nutrition and sustainable livelihood outcomes. The roadmap for promoting NUS could also apply to the 14 other countries in southern Africa that share a similar context, agro-ecologies, culture and heritage with South Africa.

## Author contributions

TM and AM had the original idea for the article and carried out the conceptualization of the article. TC did the initial data collection. TM and VC then led the write-up of the manuscript and all co-authors read and revised the manuscript.

### Conflict of interest statement

The authors declare that the research was conducted in the absence of any commercial or financial relationships that could be construed as a potential conflict of interest.
